# Exercise ameliorates anxious behavior and promotes neuroprotection through osteocalcin in VCD‐induced menopausal mice

**DOI:** 10.1111/cns.14324

**Published:** 2023-07-04

**Authors:** Yiting Kang, Jie Yao, Xiaohang Gao, Hao Zhong, Yifei Song, Xiaohui Di, Zeguo Feng, Lin Xie, Jianbao Zhang

**Affiliations:** ^1^ Key Laboratory of Biomedical Information Engineering of Ministry of Education, Institute of Health and Rehabilitation Science, School of Life Science and Technology Xi'an Jiaotong University Xi'an China; ^2^ School of Nursing Shaanxi University of Chinese Medicine Xianyang China

**Keywords:** anxiety, apoptosis, exercise, menopause, osteocalcin, VCD

## Abstract

**Aims:**

As the ovaries age and women transition to menopause and postmenopause, reduced estradiol levels are associated with anxiety and depression. Exercise contributes to alleviate anxiety and depression and the bone‐derived hormone osteocalcin has been reported to be necessary to prevent anxiety‐like behaviors. The aim of this study was to investigate the effects of exercise on anxiety behaviors in climacteric mice and whether it was related to osteocalcin.

**Methods:**

Menopausal mouse model was induced by intraperitoneal injection of 4‐vinylcyclohexene diepoxide (VCD). Open field, elevated plus maze, and light–dark tests were used to detect anxious behavior in mice. The content of serum osteocalcin was measured and its correlation with anxiety behavior was analyzed. BRDU and NEUN co‐localization cells were detected with immunofluorescence. Western blot was applied to obtain apoptosis‐related proteins.

**Results:**

The VCD mice showed obvious anxiety‐like behaviors and 10 weeks of treadmill exercise significantly ameliorated the anxiety and increased circulating osteocalcin in VCD mice. Exercise increased the number of BRDU and NEUN co‐localization cells in hippocampal dentate gyrus, reduced the number of impaired hippocampal neurons, inhibited the expression of BAX, cleaved Caspase3, and cleaved PARP, promoted the expression of BCL‐2. Importantly, circulating osteocalcin levels were positively associated with the improvements of anxiety, the number of BRDU and NEUN co‐localization cells in hippocampal dentate gyrus and negatively related to impaired hippocampal neurons.

**Conclusion:**

Exercise ameliorates anxiety behavior, promotes hippocampal dentate gyrus neurogenesis, and inhibits hippocampal cell apoptosis in VCD‐induced menopausal mice. They are related to circulating osteocalcin, which are increased by exercise.

## Graphical abstract

Treadmill exercise ameliorated anxiety behavior, promoted hippocampal dentate gyrus neurogenesis, and inhibited hippocampal cell apoptosis in VCD‐induced menopausal mice. They are related to circulating osteocalcin, which are increased by exercise.

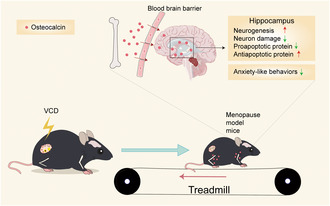

## 1 INTRODUCTION

Perimenopause is a midlife transition period for women from the reproductive period to the nonreproductive period. It comes with a complex hormonal environment change and an elevated risk of emotional disorders. Women's Health Across the Nation (SWAN) showed that the anxiety of perimenopausal women increased from 4.6% to 13.5% compared with premenopausal women.^1^ Moreover, neurological perimenopausal symptoms, including anxiety and depression can cause structural damage to the brain and other organs, particularly increasing the incidence of neurodegenerative diseases in later life.^2^, ^3^ Estrogen replacement therapy is used as an effective treatment for perimenopausal anxiety. However, a large amount of evidence confirms that long‐term estrogen therapy increases the risk of breast and ovarian cancer, stroke, and cardiovascular disease.^4^, ^5^, ^6^ Fortunately, there is growing evidence that physical activity is effective to prevent and relieve anxiety and depression along with avoiding the side effects of medication.^7^ However, the mechanism underlying the anxiolytic effects of exercise, particularly on menopausal, are still unclear.

Osteocalcin, a hormone derived from the osteoblasts, is highly studied for its function in regulating brain activity. There exist two forms of osteocalcin: carboxylated osteocalcin (cOC) and undercarboxylated osteocalcin (ucOC). The cOC is inactive and is mainly stored in the bone matrix to maintain the normal mineralization process of bone and ucOC will enter the bloodstream and triggers a wide range of tissue biological activities.^8^ In recent years, the effects of ucOC on energy metabolism and brain development were intensively revealed.^9^, ^10^, ^11^, ^12^ It was found that osteocalcin can cross the blood–brain barrier and direct bind to neurons in the brainstem, midbrain, and hippocampus, preventing anxiety and depression and improving learning and memory.^11^ Peripheral delivery of osteocalcin for 2 months was sufficient to improve memory and reduce anxiety‐like behavior in 16‐month‐old mice.^9^ We are interested in whether exercise, which is effective in promoting bone health,^13^ can influence osteocalcin production and thus improve anxiety behavior in menopausal mice.

The hippocampus is an important structure of the limbic system in the brain and it is closely related to emotional responses such as anxiety.^14^ An earlier study reported that hippocampal neuronal apoptosis and neuroinflammation are associated with anxiety and depression.^15^ The hippocampus is also one of the main brain regions affected by E2. Application of an estrogen receptor antagonists to the hippocampus increased anxiety and depression in naturally estrous female rats, while hippocampus or subcutaneous E2 injection in ovariectomized rats reduced anxiety and depression.^16^

In this study, we tested the effects of exercise on anxiety in VCD‐induced ovarian atrophy mice by open‐field test (OFT), elevated plus‐maze test (EPMT), and light/dark test (LDT). Furthermore, circulating osteocalcin and neuronal apoptosis and neurogenesis were evaluated to reveal the possible mechanisms.

## 2 MATERIALS AND METHODS

### 2.1 Animals

Forty‐four mature female C57BL/6J mice aged 56–62 days were purchased from Charles River Laboratories (Beijing, China). The mice were housed at a density of 3–4 per cage in a well‐ventilated, pathogen‐free environment with a temperature of 22 ± 1°C and a relative humidity of 45%–50%. The mice had ad libitum access to food and water during rearing. All procedures were approved by the Animal Care and Use Committee of Xi'an Jiaotong University and were performed in accordance with the National Institutes of Health Guide for the Care and Use of Laboratory Animals (NIH Publication no. 8023, revised 1978).

### 2.2 VCD mouse model of menopause and exercise treatment

**FIGURE 1:**
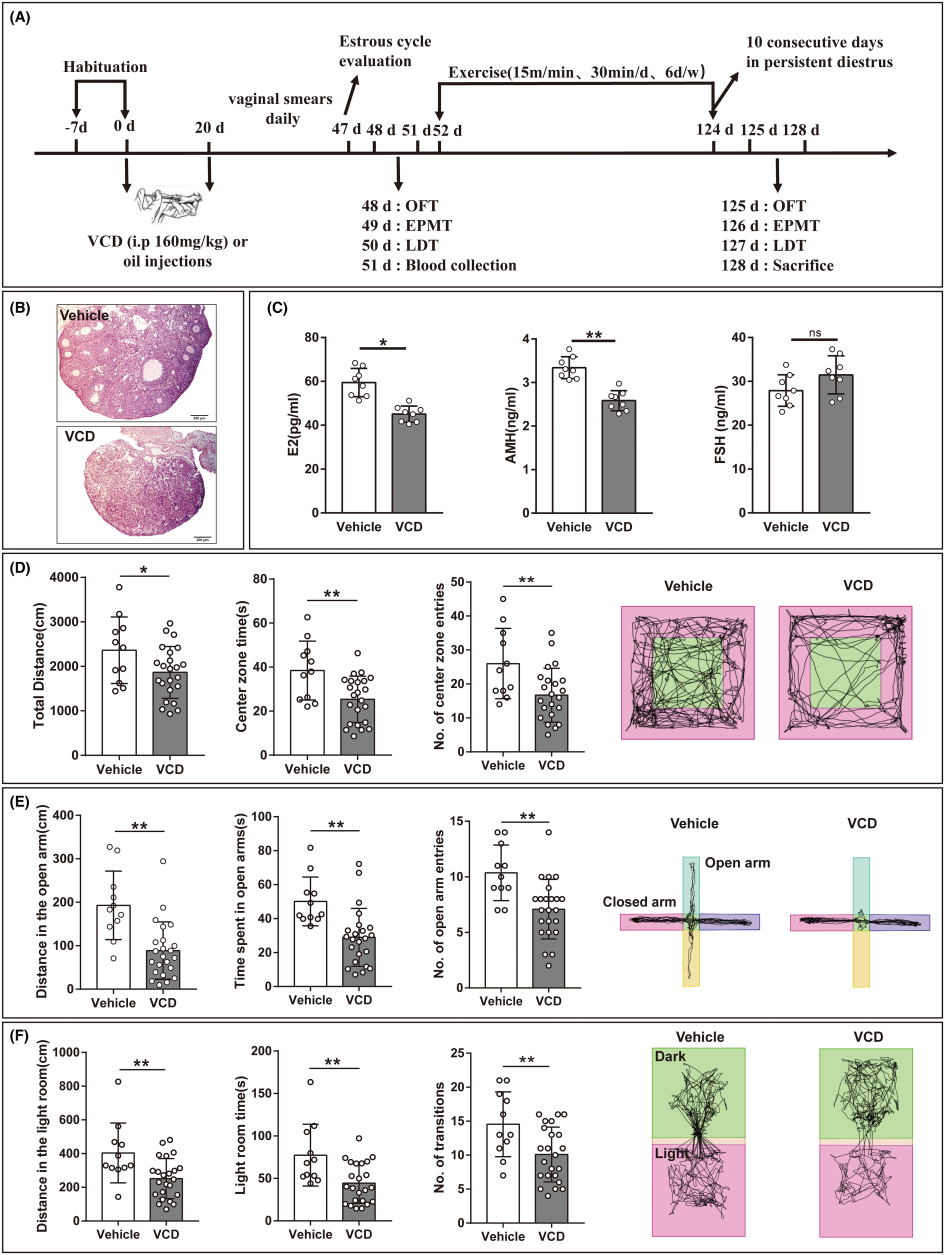
VCD mice showed anxiety‐like behaviors. (A) Experimental timeline. (B) Representative pictures of ovaries stained by HE in vehicle mice and VCD mice. Scale bars = 200 μm. (C) serum estradiol (E2), anti‐Muller hormone (AMH), and follicle stimulation hormone (FSH) in vehicle mice and VCD mice (*n* = 8, Student's *t*‐test for E2 and AMH, Mann–Whitney *U* test for FSH). (D) Total distance, center zone time, NO. of center zone entries in OFT and representative motion trajectories of mice in the OFT, the green area in the middle is the central zone (25 cm × 25 cm) (*n* = 11 or 23, Student's *t*‐test for total distance, Mann–Whitney *U* test for the center zone time and NO. of center zone entries). (E) Distance in the open arm, open arm time, No. of open arm entries in EPMT, and representative motion trajectories of mice in the EPMT, vertical green and yellow areas are open arms, horizontal pink and purple areas are closed arms (*n* = 11 or 23, Mann–Whitney *U* test). (F) Distance in the light room, light room time, No. of transitions in the LDT, and presentative motion trajectories of mice in the LDT, the green area is the dark room and the pink area is the light room (*n* = 11 or 23, Student's *t*‐test for No. of transitions, Mann–Whitney *U* test for the distance in the light room and light room time). *p < 0.05, **p < 0.01, and ns means no significance.

After 1 week of acclimatization, the mice were randomly divided into Vehicle group (*n* = 16) and VCD group (*n* = 28) by completely random method. Mice in the VCD group were intraperitoneally injected with 160 mg/kg, 2.5 μL/g VCD (Sigma‐Aldrich). The same volume of corn oil was injected into the Vehicle group and all mice were injected continuously for 20 days.^17^ After the intraperitoneal injection was completed, the estrus cycle was measured by vaginal smear at the same time every day. The normal estrus cycle of mice was 4–5 days, and two mice with irregular estrus cycle were excluded. The estrus cycle of the other mice was 4–5 days at the beginning of monitoring. At 47 days after the first injection of VCD, the estrus cycles of VCD group mice were extended to 6–7 days and 4 mice (one cage) were sacrificed in each group. Behavioral tests were performed on other mice to detect whether the disrupted estrus cycle mice exhibited anxiety‐like behaviors. After the behavioral tests, blood was collected from the submandibular venous plexus, and the mice in the VCD group were randomly divided into the VCD control group (VCD, *n* = 11) and the VCD combined exercise intervention group (VCD + EX, *n* = 12) by operators who were unaware of the experimental design. On the basis of the previous literature,^18^, ^19^ the exercise was set as 15 m/min, 30 min/day, and 6 days/week. The intensity was approximately 80% of the VO_2max_ determined in previous studies.^20^ The mice were trained for 3 days and then begin to exercise experiments. All exercises were conducted between 8:00 and 10:00 p.m. Until the mice were in diestrus for more than 10 consecutive days and the mice were judged acyclic,^21^ the exercise intervention was stopped. All mice were sacrificed by intraperitoneal injection of 100 mg/kg of pentobarbital sodium at a concentration of 1% after behavioral testing. In this study, mice showed disrupted estrus cycles on the 47th day and acyclicity on the 124th after the onset of VCD injection. The specific experimental process is shown in Figure 1A.

### 2.3 Morphometric analysis

Mice were deeply anesthetized by intraperitoneal injection of 100 mg/kg of pentobarbital sodium at a concentration of 1% and transcardially perfused with 4% paraformaldehyde. The brain and ovaries were fixed in 4% paraformaldehyde for 48 h separately and then dehydrated and embedded in paraffin. Subsequently, the wax was fixed on an ultra‐thin semi‐automatic microtome (Leica). After fixation, serial sections of 4 μm were adhered to anti‐shedding slides and dried in a baking table at 60°C. Finally, the glass slides were stained with hematoxylin and eosin. Histophysiological examined using light microscopy (Nikon) to estimate the extent of hippocampal neuron damage and ovarian follicular atresia.

### 2.4 Blood collection and serum analysis

Serum samples in this study were collected in two ways. Blood was collected from the submandibular venous plexus on the 51st day after the first VCD, and was collected from the abdominal aorta on the 128th day. The mice were be anesthetized, respectively, with 2% isoflurane and pentobarbital sodium (100 mg/kg) during collecting blood. Commercial ELISA kits were used to detect serum estradiol (E_2_, Cloud‐Clone), anti‐Mullerian hormone (AMH; Cloud‐Clone), follicle‐stimulating hormone (FSH; Cloud‐Clone), Gla‐Osteocalcin (cOC; Takara), Glu‐Osteocalcin (ucOC, Takara) to monitor hormone levels in mice and the detection procedures were strictly followed in accordance with the instructions. Absorbance was measured on a model 680 microplate reader (Bio‐Rad Corp). Detection limits were 12.35 pg/mL (E_2_), 123.5 pg/mL (AMH), 2.47 ng/mL (FSH), 0.5 ng/mL (cOC) and 0.25 pg/mL (ucOC).

### 2.5 Behavior analysis

Open‐field test (OFT), elevated plus maze (EPMT), and light–dark test (LDT) were used to detect the anxiety of mice in each group. The behavioral phenotypes of mice were detected in three consecutive days (one test per day). Mice were acclimated to the behavioral testing laboratory for at least 3 hours before formal behavioral tests.

#### 2.5.1 Open‐field test

The open field is a square, white box with dimensions 50 cm × 50 cm × 40 cm. Mice were gently placed in the center of the arena and allowed to freely explore for 5 min in a lighting environment (approximately 120 lux).^22^ Before each trial, the box was cleaned using 30% ethanol. The experimenter leaves the room after placing the animals in the arena. All trials were recorded by video in top‐view and analyzed with Labmaze V3.0 tracking software (Beijing Zhongshidichuang). The distance moved in the central area (25 × 25 cm square), the time spent in the central, and the total distance traveled were collected to evaluate anxiety levels.

#### 2.5.2 Elevated plus maze test

EPMT is a widely used classical test to evaluate anxiety in rodents.^23^ The EPMT consisted of a pair of open arms (30 cm × 6 cm) and a pair of closed arms (30 cm × 6 cm × 15 cm) 65 cm above the ground and connected by a central region (6 cm × 6 cm). The two arms were at a 90° angle. At the beginning of the experiment, the mice were placed in the central area facing the open arm and were allowed to move freely in the maze for 5 min. Total distance, time, and number of entries to the open arms were recorded and analyzed using Labmaze V3.0. Mice were removed at the end of the experiment and both arms were cleaned with 30% alcohol.

#### 2.5.3 Light/dark test

The LDT tests were carried out according to the previously reported protocol.^24^ In brief, the instrument consists of a black box and a transparent box of equal size (15 cm × 15 cm × 28 cm) connected by a hole in the middle (5 cm × 4 cm). The box was placed on a 40 cm × 40 cm × 50 cm infrared visual equalizer box. There is an infrared camera above the box to detect the movement of the tested animals. The mice were gently placed in the center of the bright box, facing the dark box, and allowed them to move freely in the device for 5 min. Total distance, time, and number of entries to the lit compartment were recorded and analyzed using Labmaze V3.0. The chamber was scrubbed with 30% alcohol at the end of each experiment.

### 2.6 BRDU administration

BRDU (5‐bromo‐2‐deoxyuridine) is used to detect the proliferation of hippocampal neural stem cells. BRDU immunofluorescence is a tool to confirm adult neurogenesis in the nervous system.^25^ After exercise, 10 mg/mL BRDU (Sigma‐Aldrich) solution was prepared, filtered, and sterilized, 5–6 mice in each group were given intraperitoneally at a dose of 50 mg/kg for 4 times, with an interval of 4 h.^11^ The brain was perfused 24 h after the First dose. The proliferation of hippocampal neural stem cells hippocampus was detected by BRDU immunofluorescence staining.^11^

### 2.7 Immunofluorescent

The coronal sections of the hippocampus were incubated with 3% BSA to block nonspecific binding for 1 h, followed by an overnight incubation with the anti‐NEUN (1:3000, GB11138; Servicebio), anti‐BRDU (1:500, GB12051; Servicebio) at 4°C, respectively. On the next day, the secondary antibodies were tagged with goat anti‐rabbit Cy3 (1:500, GB21303; Servicebio), goat anti‐mouse Alexa 488 (1:500, GB25301; Servicebio) for 1 h. Then slices were stained with 4′,6‐diamidino‐2‐phenylindole (DAPI, 0.5 μg/mL, 10 min, Servicebio). After staining, the sections were treated with autofluorescence quenching and then the sections were sealed with anti‐fluorescence quenching. The sections were scanned using a digital section scanner (Panoramic DESK P250, 3DHISTECH). NEUN staining images were captured from the hippocampus at 4 × 10 magnification, from the CA1, CA3, and DG regions at 20 × 10 magnification. BRDU staining images were captured from DG at 20 × 10 magnification.

### 2.8 Western blot analysis

The Western blot procedure was carried out according to the methods of a previous study.^26^ Briefly, hippocampal proteins were extracted using RIPA total protein lysates (Beyotime) supplemented with a mixture of protease and phosphatase inhibitors (Beyotime). The total protein concentration was quantified using the BCA protein quantification kit (Beyotime). Equal amounts of total protein (30 μg) were separated on 8%–15% polyacrylamide gels and transferred onto polyvinylidene fluoride membrane (Millipore) using Trans‐Blot Turbo (Liuyi). After blocking with a 5% defatted milk powder solution in 0.1% TBST for 1 h at room temperature, the membranes were incubated with primary antibodies overnight at 4°C followed by incubation with the corresponding secondary antibodies at room temperature for 1 h. The blots were visualized with ECL‐plus reagent (Millipore), and the bands' density were quantified by Image J analysis software. The primary antibodies were used: BAX (1:800, 2772S), Caspase‐3 (1:800, 9662S) and cleaved Caspase‐3 (1:500, 9664T) from Cell signaling technology; BCL‐2 (1:2000, 26593‐1‐AP), NEUN (1:1000, 26975‐1‐AP) from Proteintech and PARP (1:800, T40050) from Abmart.

### 2.9 Statistical analysis

All data were tested by one‐sample Kolmogorov–Smirnov test to verify whether the data were normally distributed. Regarding normally distributed data, two‐tailed unpaired Student's t‐test was used to determine the differences between two groups, and One‐way ANOVA was employed to evaluate the existence of differences among the three groups. Once a significant difference was detected, Tukey's multiple comparison test was performed to determine the significance between every two groups. For nonnormally distributed data, the Mann–Whitney *U* test was applied to determine the differences between two groups, and Kruskal‐Wallis One‐way ANOVA was employed to evaluate the existence of differences among the three groups, all pairwise was selected for multiple comparisons. The data were expressed as mean ± SD. *p* Values of 0.05 and 0.01 were regarded as significant. All statistical analyses were conducted using SPSS 25.0 (IBM SPSS Statistics v.25) software and GraphPad Prism 7.0 (GraphPad Software).

## 3 RESULTS

### 3.1 Ovarian follicles and serum hormones changed in VCD mice

In order to examine the development of ovarian follicles and serum hormone levels in VCD mice with disrupted estrus cycles, we performed HE staining on mouse ovaries and detected serum E_2_, AMH, and FSH levels. As shown in Figure 1B, there were primary follicles, secondary follicles, sinus follicles, corpus luteum, and a small number of atresia follicles in the ovaries of Vehicle mice. However, in the ovaries of VCD mice, a lot of atresia follicles appeared. As illustrated in Figure 1C, serum E_2_ and AMH in VCD mice were significantly decreased compared with the Vehicle group, while follicle stimulation hormone was not significantly changed (*p* = 0.46). The results indicated that VCD‐induced menopausal mouse model was successful.

### 3.2 VCD mice showed anxiety‐like behaviors

Anxiety‐like behaviors were analyzed by three conflict‐based tests, including OFT, EPMT, and LDT. As shown in Figure 1D, VCD mice showed a dramatic decrease in the total distance moved and the time spent in center. In addition, the number of VCD mice entering the center was also significantly reduced compared with Vehicle mice. In the EPMT, anxiety‐related behavior is defined by a lesser time spent, shorter distance moved in the open arms, and fewer entries to the open arms, which was observed in VCD mice as shown in Figure 1E. In the LDT, rodents innately dislike brightly lit areas. As illustrated in Figure 1F VCD mice displayed fewer distances moved and spent less time in the lit compartment. They also made fewer transitions between compartments. Our results indicated that VCD mice showed an obvious anxiety‐like behaviors.

### 3.3 Exercise did not affect the levels of ovarian follicles and serum hormones in VCD mice

**FIGURE 2:**
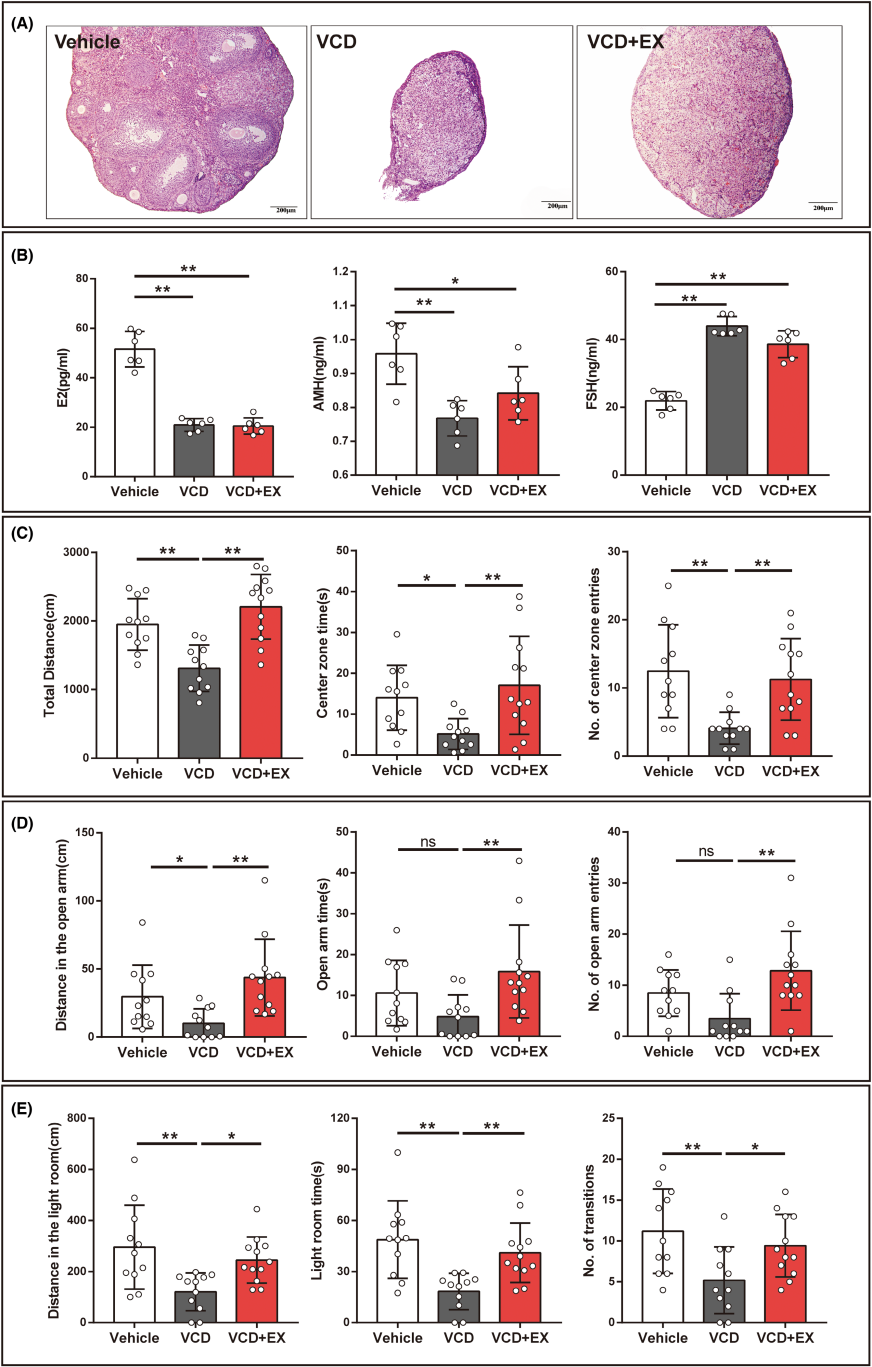
Exercise ameliorated anxiety‐like behaviors in VCD mice. (A) Representative picture of ovaries stained by HE. (B) Serum estradiol (E2), Serum anti‐Muller hormone (AMH), Serum follicle stimulation hormone (FSH) (*n* = 6, Kruskal‐Wallis One‐way ANOVA for E_2_ and AMH, One‐way ANOVA for FSH). (C) Total distance, center zone time, No. of center zone entries in the OFT (*n* = 11 or 12, Kruskal‐Wallis One‐way ANOVA for total distance, One‐way ANOVA for center zone time and No. of center zone entries). (D) Distance in the open arm, open arm time, and No. of open arm entries in the EPMT (*n* = 11 or 12, Kruskal‐Wallis One‐way ANOVA for distance in the open arm, One‐way ANOVA for open arm time and No. of open arm entries). (E) Distance in the light room, light room time, No. of transitions in the LDT (*n* = 11 or 12, One‐way ANOVA for distance in the light room, light room time, No. of transitions). *p < 0.05, **p < 0.01, and ns means no significance.

Exercise intervention was performed when mice showed prolonged estrous cycles, and the effects of exercise on ovarian follicles and serum hormone levels were detected when mice appeared in the diestrum for more than 10 consecutive days. As shown in Figure 2A, there was almost no healthy follicle in VCD mice, while exercise had no significant effect on the status of ovarian follicles in VCD + EX group mice. With ovarian follicle atresia recession, the serum hormone levels have undergone significant changes. As illustrated in Figure 2B, the levels of serum E_2_ and AMH decreased significantly in VCD and VCD + EX mice compared with vehicle mice. While FSH significantly increased in both VCD and VCD + EX group compared with the Vehicle group.

### 3.4 Exercise ameliorated anxiety‐like behaviors in VCD mice

Vaginal smears after 10 consecutive days in diestrus indicated that the ovarian follicular reserve of the mice was depleted and the ovaries were senescent. The effect of exercise on anxiety‐like behavior in mice was observed with behavioral experiments. As shown in Figure 2C, in the OFT, VCD mice showed a drastic decrease in the total distance moved, the time spent in the center, and center entries compared with Vehicle mice. While the VCD + EX group mice significantly increased the total distance in the open field, the time spent in the center, and center entries after 10 weeks of aerobic running. As illustrated in Figure 2D, in the EPMT, the distance moved in the open arm decreased in VCD mice compared with Vehicle mice, 10 weeks of exercise significantly increased the distance moved, the time spent in open arms and open arms entries in VCD + EX mice. As illustrated in Figure 2E, in the LDT, compared with Vehicle mice, VCD mice displayed fewer distances moved and less time in the lit compartment, but they also made fewer transitions between compartments. As was expected, exercise reversed it. The results suggested that 10‐week aerobic treadmill exercise significantly ameliorated anxiety‐like behaviors in VCD‐induced ovarian aging mice.

### 3.5 Exercise increased circulating cOC and ucOC in VCD mice

**FIGURE 3:**
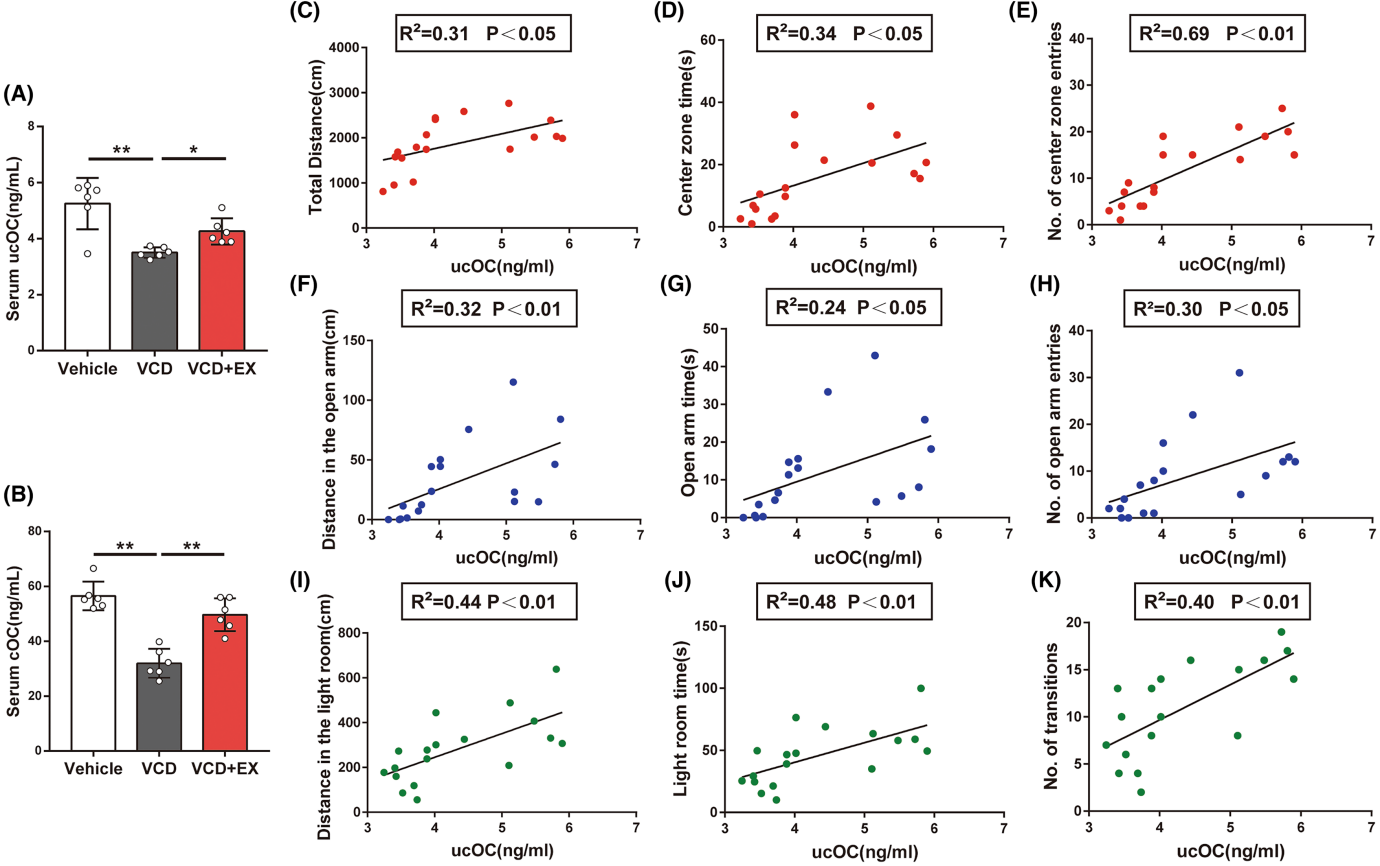
Exercise increased circulating cOC, ucOC in VCD mice and circulating ucOC positively correlated with improved anxiety behavior in mice. (A) Serum ucOC (*n* = 6, One‐way ANOVA). (B) Serum cOC (*n* = 6, One‐way ANOVA). (C–E) Correlation analysis between serum ucOC and behavioral results in OFT (Pearson Correlation Analysis). (F–H) Correlation analysis between serum ucOC and behavioral results in EPMT (Pearson Correlation Analysis). (I–K) Correlation analysis betweeun serum ucOC and behavioral results in LDT (Pearson Correlation Analysis). *p < 0.05, **p < 0.01.

Considering the role of osteocalcin in the regulation of hippocampal‐dependent anxiety behavior, we measured the serum osteocalcin levels in mice after exercise. As shown in Figure 3A,B, VCD mice showed varying decreases in serum osteocalcin, including cOC and ucOC. 10 weeks of aerobic treadmill exercise significantly increased serum cOC and ucOC in VCD‐induced ovarian senescent mice.

### 3.6 Circulating ucOC levels positively correlate with improved anxiety behavior in mice

ucOC, rather than cOC, is the active form of osteocalcin, so we studied the relationship between ucOC and behavioral test results. As shown in Figure 3C–E, higher serum ucOC levels were associated with better performance in the OFT. As shown in Figure 3F–H, serum ucOC were significantly and positively correlated with the distance, time of movement, and number of entries in the open arm of the mice in the EPMT. As shown in Figure 3I–K, more serum ucOC were correlated with greater behavioral phenotypes in the LDT.

### 3.7 Exercise attenuated neural apoptosis in the hippocampus of VCD mice

**FIGURE 4:**
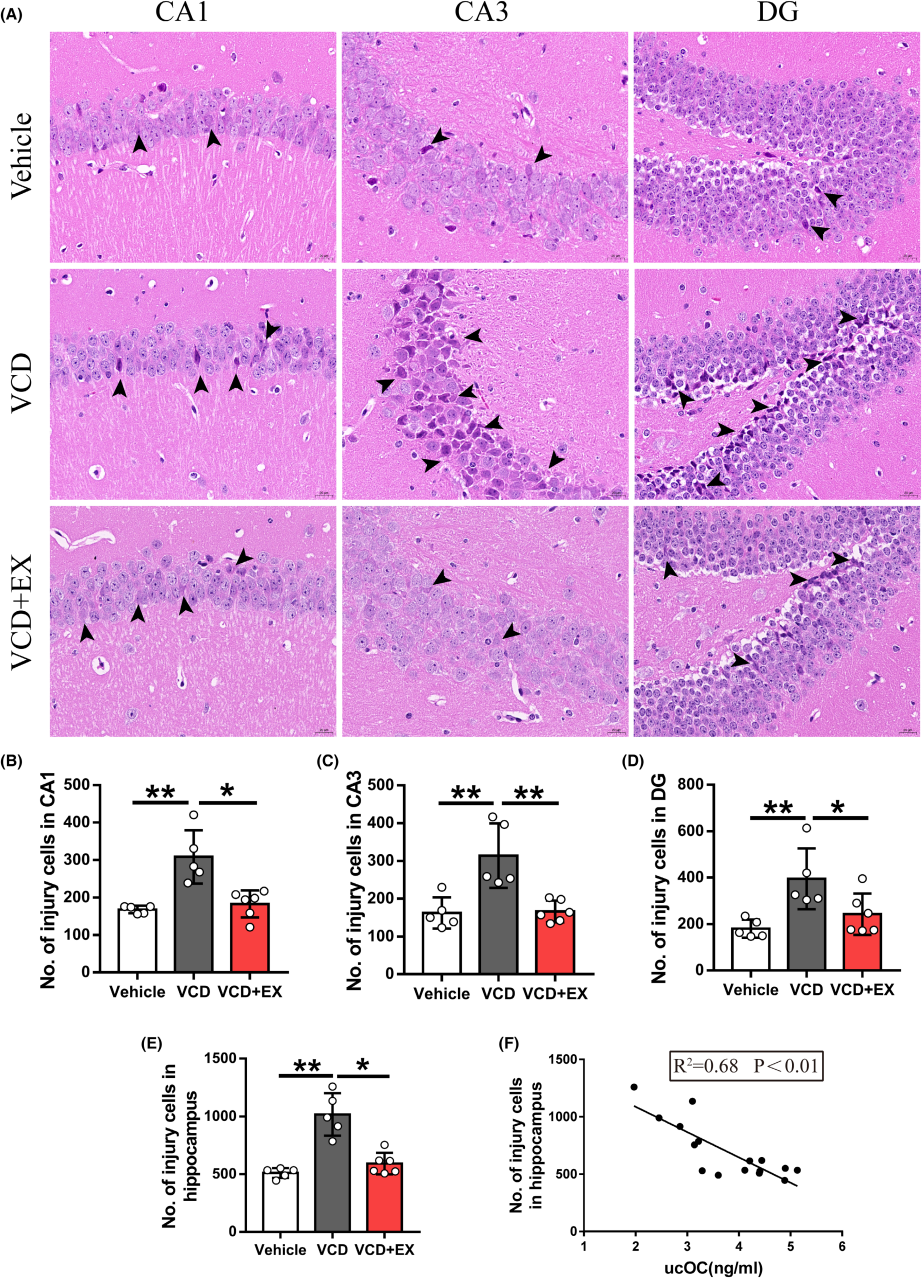
Exercise attenuated neural injury in the hippocampus of VCD mice. (A) Representative pictures of hippocampus HE staining (Scale bar: 200 μm). (B–E) The number of injury neuron cells in the hippocampus, CA1, CA3, DG (*n* = 5 or 6, One‐way ANOVA). (F) Correlation analysis between serum ucOC and the number of injury neuron cells in the hippocampus (Pearson Correlation Analysis). *p < 0.05, **p < 0.01.

The pathological changes of hippocampal neurons were examined by hematoxylin‐eosin staining. As shown in Figure 4A, the hippocampus in the VCD group exhibited a greater number of pyramidal cells with shrunken or irregular shapes and deeper staining. Exercise attenuated the degree of injured cells (shrunken or irregular shape and deeper staining). Quantitative analysis revealed that the number of injured cells in the hippocampal, including CA1, CA3, and DG regions, were significantly decreased with exercise (Figure 4B–E). Moreover, the number of injured cells in the hippocampal was negatively correlated with osteocalcin (Figure 4F, *R* ^2^ = 0.68, *p* < 0.01).

**FIGURE 5:**
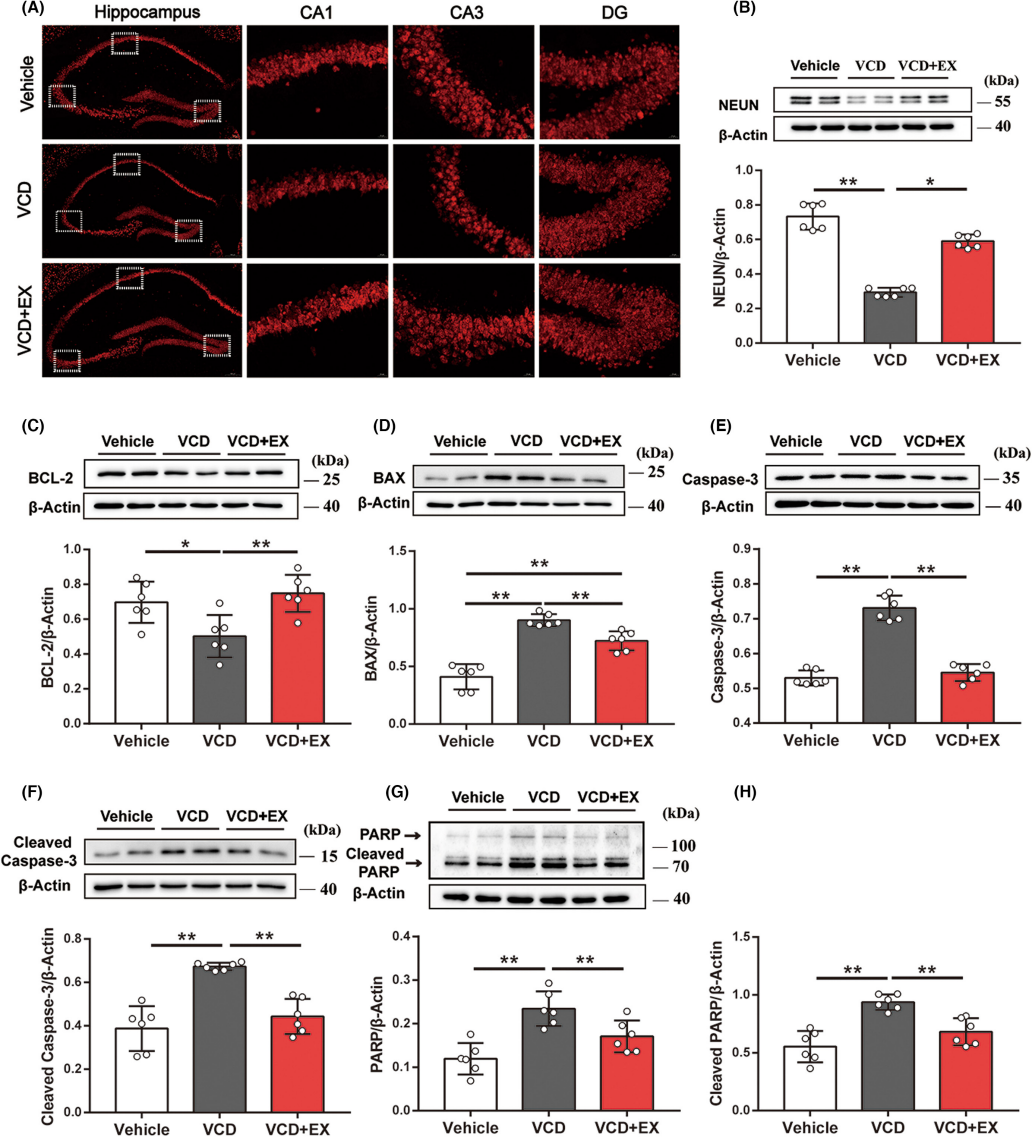
Exercise attenuated neuron loss and inhibited hippocampal cell apoptosis. (A) Representative pictures of hippocampus NEUN staining (Scale bar: 200 μm or 50 μm). (B) NEUN protein expression in mouse hippocampus (*n* = 6, Kruskal‐Wallis One‐way ANOVA). (C) The BCL‐2 protein expression in mouse hippocampus (*n* = 6, One‐way ANOVA). (D) The BAX protein expression in mouse hippocampus (*n* = 6, One‐way ANOVA). (E) The Caspase‐3 protein expression in mouse hippocampus (*n* = 6, Kruskal‐Wallis One‐way ANOVA). (F) The cleaved caspase‐3 protein expression in mouse hippocampus (*n* = 6, One‐way ANOVA). (G) The PARP protein expression in mouse hippocampus(*n* = 6, One–way ANOV). (H) The cleaved PARP protein expression in mouse hippocampus (*n* = 6, One‐way ANOVA). *p < 0.05, **p < 0.01.

Immunofluorescence staining of NEUN (a marker of mature neuron) was performed in order to examine the density of neurons in the hippocampus. As illustrated in Figure 5A,B, hippocampal NEUN‐positive neuron and NEUN protein expression decreased in the VCD group compared with the Vehicle group, while exercise alleviated the decrease in NEUN‐positive neuron and NEUN protein expression in the VCD + EX group (*p* < 0.01). The findings suggested that exercise could attenuate neuronal damage in VCD‐induced ovarian aging mice.

Apoptosis and the underlying molecular mechanisms of cell death and survival are considered the primary mechanisms responsible for anxiety and depression.^27^ Therefore, we evaluated the effects of exercise on apoptosis‐related proteins in the hippocampus of mice. The expression of antiapoptotic and proapoptotic proteins of the BCL‐2 family is one of the key regulatory steps in the regulation of apoptosis, and they determine the sensitivity to apoptosis, with BCL‐2 and BAX being the most representative.^28^ As shown in Figure 5C,D, hippocampal BAX expression significantly increased in VCD mice compared to Vehicle group mice, while BCL‐2 significantly decreased. However, exercise significantly promoted the expression of the antiapoptotic protein BCL‐2 and inhibited the expression of the proapoptotic protein BAX. Since Caspase‐3 is considered as the ultimate executor of caspase family‐mediated apoptosis, activation of Caspase‐3 is considered as an indicator of the apoptotic process.^29^ As shown in Figure 5E,F, hippocampal Caspase‐3 and cleaved Caspase‐3 protein expression significantly increased in VCD mice compared to Vehicle mice, while exercise significantly inhibited their expression. The expression of PARP and cleaved PARP (Figure 5G,H), the cleavage substrates of cleaved Caspase3, increased in the hippocampus of VCD mice, which was inhibited by exercise. The results suggested that exercise can inhibit the hippocampal cell apoptosis of VCD‐induced ovarian senescent mice.

### 3.8 Exercise increased the number of BRDU‐positive cells in hippocampal dentate gyrus

**FIGURE 6:**
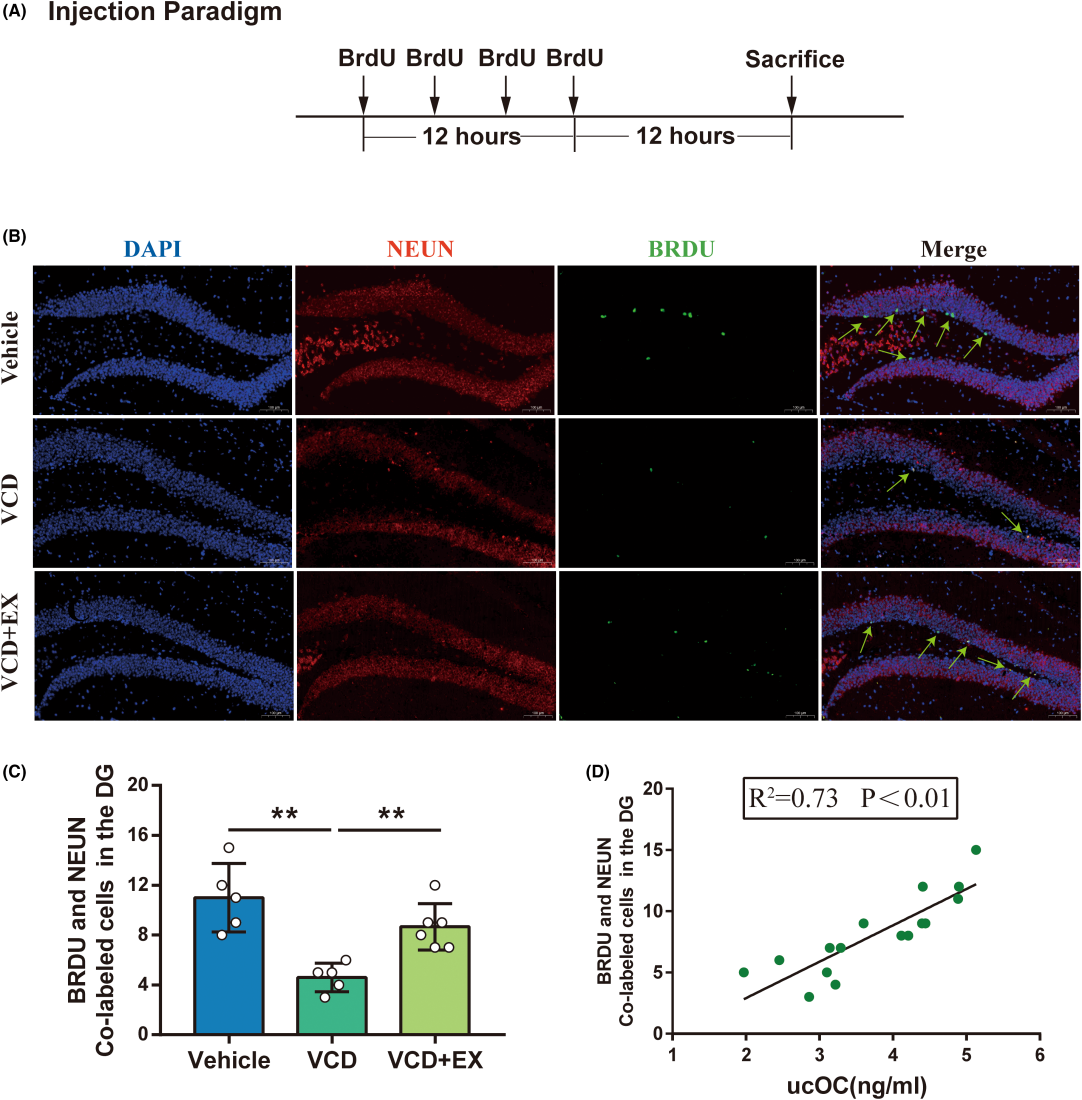
Exercise increased the number of BRDU and NEUN co‐localization cells in hippocampal dentate gyrus. (A) BRDU injection paradigm. (B) Representative pictures of BRDU (green) and NEUN (red) immunofluorescence staining (the arrow indicates NEUN and BRDU Co‐labeled cells, Scale bar: 100 μm). (C) Total number of BRDU and NEUN co‐localization cells in the DG (*n* = 5 or 6, One–way ANOVA). (D) Correlation analysis between serum ucOC and BRDU and NEUN co‐labeled cells in the DG (Pearson Correlation Analysis). *p < 0.05, **p < 0.01.

Adult hippocampus continuously generates new neurons, which are functionally integrated into hippocampal circuits and contribute to memory and mood regulation.^30^ Accumulating evidence shows that impaired adult hippocampal neurogenesis results in anxiety and depression in both animal models and humans.^30^, ^31^, ^32^ To determine whether the anxiety‐like behavior was associated with the impairment of adult neurogenesis in the hippocampus, BRDU staining of menopausal mice was performed. As illustrated in Figure 6B, changes in neurogenesis in the dentate gyrus were assessed by comparing the number of BRDU‐positive cells in the hippocampal dentate gyrus. The quantitative analysis showed that the number of BRDU and NEUN co‐localization cells in VCD mice significantly decreased, compared with Vehicle mice, and the exercise significantly increased the number of BRDU^+^ and NEUN^+^ cells in VCD + EX mice (Figure 6C). Moreover, the number of BRDU and NEUN co‐localization cells was positively correlated with osteocalcin (Figure 6D, *R* ^*2*^ = 0.73, *p* < 0.01). These results showed that exercise ameliorates impaired adult neural stem cell proliferation of VCD mice and it was likely dependent on the osteocalcin level.

## 4 DISCUSSION

Menopause is a natural physiological process of women, over 46% of women aged 45–55 suffer from anxiety and depressive symptoms during or after menopause.^33^ Exercise has a positive effect on various complications related to mood disorders such as anxiety and depression. In the study, we confirmed that exercise effectively ameliorated anxiety behavior and increased circulating osteocalcin levels in VCD‐induced ovarian senescent mice. Importantly, the improvement of anxiety behavior, increases of BRDU and NEUN co‐localization cells and suppression of the hippocampal neuron degeneration in VCD mice induced by exercise was all positively correlated with circulating osteocalcin level in serum.

Some of the studies showed that 4‐vinylcyclohexene diepoxide (VCD) directly acts on the primitive and primary follicles of the ovary and was nontoxic to non‐ovarian tissues.^34^ In our study, menopausal mouse models with follicle failure were successfully induced by intraperitoneal injection of VCD into C57BL/6J mice for 20 days. The model simulated progressive ovarian failure in menopausal women, including the transition from perimenopause to menopause. After VCD injection, numerous atretic follicles were found in the ovary of mice and then almost disappeared. The hormone levels of VCD mice also changed with the apoptosis of ovarian follicles. AMH was one of the most biologically meaningful biomarkers related to ovarian reserve.^35^ It was produced in granulosa cells of the primary to small sinus follicles of the adult ovary.^35^ Kevenaar et al.^36^ found that the AMH decrease in blood as mice age was closely related to the number of primary and growing follicles. In our study, serum E2 and AMH in VCD mice decreased at days 51st and 128th after the first VCD injection. However, FSH did not change at 51th after the first VCD administration but increased on the 128th day. It was known that in women, FSH levels may not change in the early perimenopausal period and rise in the later period.^37^ Previous studies in rats showed that VCD was injected at 80 mg/kg for 30 consecutive days, FSH level did not change at the 60th day after the first injection and was significantly higher than that of the control group after 120 days.^38^ Therefore, we believe that the period tested for the first time in this study (51th day after the first injection of VCD) was early perimenopausal, when FSH levels were not significantly elevated. In fact, a decrease in AMH seems to be a more reliable biomarker for evaluating menopause than a change in FSH concentration. Consistent with our results, mice were intraperitoneally injected with VCD for 20 consecutive days, on the 37th day after injection, the serum estrogen and AMH levels decreased significantly.^39^ When B6C3F1 female mice are treated with VCD for 10 consecutive days, the decline of E2 levels occurs by approximately day 110 after the onset of VCD administration.^34^ These results, including the measurement of estrous cycle, ovarian follicle count, and serum hormone levels, indicated that we have successfully constructed a menopausal mouse model. In the study, VCD‐induced mice exhibited high anxiety‐like behaviors when the estrus cycle disrupted compared with Vehicle mice. Our results further support that the VCD‐induced reproductive aging mouse model was a reliable model for studying mood disorders of menopausal women.

Exercise is a promising, affordable, and easily accessible non‐pharmacological method to improve anxiety disorders. Aerobic exercise three times a week for 4 weeks improved psychological stress and anxiety symptoms in anxious patients from the community.^40^ Twelve weeks of Pilates exercise improved sleep quality, anxiety, and depression in Spanish postmenopausal women 60 years old and over.^41^ Moreover, 10 days of moderate‐intensity treadmill exercise improved anxiety‐like behavior and promoted the neurogenesis in mice with traumatic brain injury.^42^ In the study, consistent and regular moderate treadmill exercise for the VCD mice was performed from the onset of estrous cycle prolongation until to acyclic. Exercise significantly attenuated anxiety‐like behavior of VCD mice. Our results indicated that the modes and intensity of exercise used in the study were effective.

Serum osteocalcin is negatively associated with the risk of metabolic disorders, fasting blood glucose, glycated hemoglobin, and nonalcoholic fatty liver disease in peri‐ and postmenopausal women.^43^, ^44^, ^45^ OC^−/−^ mice showed significant anxiety and depression‐like behaviors, which were significantly favored following peripheral delivery of osteocalcin.^11^ Moreover, peripheral delivery of osteocalcin for two months reduced anxiety‐like behavior in aged female mice.^9^ In this study, exercise significantly increased serum osteocalcin levels in VCD‐induced ovarian senescent mice, and serum osteocalcin was correlated with reduced anxiety‐like behaviors. These results indicated that increased osteocalcin by exercise may contribute to the improved anxiety behaviors in female VCD mice. Clinical studies showed that 8 months of water aerobics increased functional autonomy and serum osteocalcin levels in postmenopausal women.^46^ Sixteen weeks of team handball exercise increased serum osteocalcin levels (+41.9 ± 27.0%) in postmenopausal women compared with no exercise experience.^47^ However, 10 weeks of group‐based step aerobics (GBSA) exercise had no effect on serum osteocalcin in postmenopausal women with low bone mass^48^ and 8 weeks of aerobic training decreased serum osteocalcin levels in postmenopausal women.^49^ In fact, serum osteocalcin levels exhibited a circadian rhythm, with the lowest levels in the morning and the highest levels in the evening.^50^ It may be the reason of the above inconsistent results.

Mood disorder increased apoptosis of the hippocampus neurons in patients.^51^ Hippocampal neuronal apoptosis is considered as the main factor responsible for anxiety and depression.^15^, ^27^ It was even suggested that ablation of neurogenesis may directly contribute to depression or anxiety.^52^ Estradiol plays a key role in brain development, maintaining normal brain function and protecting the brain from various neurodegenerative diseases and injuries.^53^ Removal of circulating estrogen by oophorectomy reduced the number of proliferating cells in hippocampus and it was reversed by subsequent administration of estradiol.^54^ The BCL‐2 and BAX are the key apoptosis factors and, respectively, inhibit and promote the apoptosis. They stimulate the initiator caspase and lead to the activation of the apoptosis executor Caspase‐3.^55^ Caspase‐3 constitutes an important step in apoptosis by inducing DNA degradation or cleavage.^56^ Poly (ADP‐ribose) polymerase (PARP) is one of its most important substrate proteins. After activation of apoptosis with cleaved Caspase3, cleaved PARP is formed and then perform subsequent biological functions.^57^ In this study, the expression of BAX, Caspase3, activated caspase‐3, PARP, and cleaved PARP increased in VCD mice, and the expression of BCL‐2 significantly decreased. Meanwhile, the proliferation of hippocampal nerve cells was impaired. These series of changes in neurons were mainly caused by estrogen withdrawal, which was also observed in an ovariectomized model.^58^ Treadmill exercise intervention ameliorated hippocampal cell damage, promoted hippocampal dentate gyrus neurogenesis, inhibited the expression of BAX, caspase 3, cleaved caspase‐3, PARP, cleaved PARP, and promoted the expression of BCL‐2. Our results demonstrate that treadmill exercise inhibit the apoptosis of hippocampal neurons in VCD mice through Caspase3/BCL‐2/BAX pathway. However, we did not observe any reverse changes in ovarian follicles in VCD mice with exercise, nor did we observe any changes in serum estrogen levels in mice. That means improvement of exercise on the state of hippocampal neurons in mice are independent of estrogen.

Interestingly, we detected a significant increase in serum osteocalcin levels in mice after exercise. Previous studies have shown that osteocalcin plays an integral role in brain development and the regulation of anxiety behavior in mice. Maternal osteocalcin crosses the placenta during pregnancy and improves neuronal apoptosis and neurogenesis in the embryo before it synthesizes this hormone.^11^ Intraperitoneal injection of OC can effectively mitigate 6‐hydroxydopamine (6‐OHDA)‐induced dopaminergic neuronal damage in Parkinson's rats.^59^ In addition, cellular experiments showed that osteocalcin promoted neuronal cell survival and suppressed H_2_O_2_‐induced cell death.^60^ In the study we also demonstrated that the changes of VCD mice induced by exercise were significantly related to osteocalcin level. Given the role of osteocalcin in regulating anxiety and neuronal apoptosis and considering of our results, we postulated that exercise ameliorated anxiety in VCD mice by promoting hippocampal neurogenesis and inhibiting hippocampal neuronal apoptosis, at least in part, are mediated by increasing circulating osteocalcin.

## 5 CONCLUSION

In summary, exercise can effectively promote hippocampal neurogenesis and inhibit hippocampal cell apoptosis through the Caspase3/BCL‐2/BAX pathway, and then contributes to the amelioration of anxiety behavior in menopausal mice. The effects are dependent on osteocalcin level in blood.

## AUTHOR CONTRIBUTIONS

JZ and LX designed the experiments. YK and JY conducted the experiments and performed analysis. HZ, XG, YS, and HD participated in the establishment of the animal model, exercise training, and data collection. ZF contributed analysis methods and participated in the western blot experiments. YK wrote the manuscript. JZ and LX critically revised the manuscript. All authors read and approved the final manuscript.

## CONFLICT OF INTEREST STATEMENT

The authors declare that they have no conflict of interests.

## Data Availability

The data that support the findings of this study are available from the corresponding author upon reasonable request.
